# Exposure and Risk Factors to *Coxiella burnetii*, Spotted Fever Group and Typhus Group Rickettsiae, and *Bartonella henselae* among Volunteer Blood Donors in Namibia

**DOI:** 10.1371/journal.pone.0108674

**Published:** 2014-09-26

**Authors:** Bruce H. Noden, Filippus I. Tshavuka, Berta E. van der Colf, Israel Chipare, Rob Wilkinson

**Affiliations:** 1 Department of Biomedical Science, Polytechnic of Namibia, Windhoek, Namibia; 2 Department of Microbiology, Namibia Institute of Pathology, Oshakati, Namibia; 3 Blood Transfusion Service of Namibia, Windhoek, Namibia; University of Texas Medical Branch, United States of America

## Abstract

**Background:**

The role of pathogen-mediated febrile illness in sub-Saharan Africa is receiving more attention, especially in Southern Africa where four countries (including Namibia) are actively working to eliminate malaria. With a high concentration of livestock and high rates of companion animal ownership, the influence of zoonotic bacterial diseases as causes of febrile illness in Namibia remains unknown.

**Methodology/Principal Findings:**

The aim of the study was to evaluate exposure to *Coxiella burnetii,* spotted fever and typhus group rickettsiae, and *Bartonella henselae* using IFA and ELISA (IgG) in serum collected from 319 volunteer blood donors identified by the Blood Transfusion Service of Namibia (NAMBTS). Serum samples were linked to a basic questionnaire to identify possible risk factors. The majority of the participants (64.8%) had extensive exposure to rural areas or farms. Results indicated a *C. burnetii* prevalence of 26.1% (screening titre 1∶16), and prevalence rates of 11.9% and 14.9% (screening titre 1∶100) for spotted fever group and typhus group rickettsiae, respectively. There was a significant spatial association between *C. burnetii* exposure and place of residence in southern Namibia (P<0.021). Donors with occupations involving animals (P>0.012), especially cattle (P>0.006), were also significantly associated with *C. burnetii* exposure. Males were significantly more likely than females to have been exposed to spotted fever (P<0.013) and typhus (P<0.011) group rickettsiae. Three (2.9%) samples were positive for *B. henselae* possibly indicating low levels of exposure to a pathogen never reported in Namibia.

**Conclusions/Significance:**

These results indicate that Namibians are exposed to pathogenic fever-causing bacteria, most of which have flea or tick vectors/reservoirs. The epidemiology of febrile illnesses in Namibia needs further evaluation in order to develop comprehensive local diagnostic and treatment algorithms.

## Introduction

As developing countries work to eliminate malaria, it is imperative to describe which pathogens are responsible for febrile illnesses that have historically been misdiagnosed as malaria [1]. In the Moshi area of Tanzania, arboviruses (chikungunya and dengue [2]), bacteria (*Coxiella burnetii*, spotted fever and typhus group rickettsiae) [3] and other zoonotic pathogens (leptospirosis [4], histoplasmosis [5], and brucellosis [6]) were identified as the causes of fever in patients. Studies like these are critical to support the development of diagnostic algorithms to account for other local causes of fever as malaria is eliminated [1], [7]. In Southern Africa, four countries (Namibia, Botswana, South Africa, and Swaziland) are actively engaged in malaria elimination campaigns [8].

In Namibia, the strategy to eliminate malaria has achieved infection rates close to pre-elimination levels and is on track to eliminate malaria in the country by 2020 [9]. With malaria mapped to specific ‘hot spots’, active surveillance has been implemented in the areas of highest incidence with rapid diagnostic tests (RDT) required for all fever cases [10]. The RDT results indicate that almost 90% of the fever cases in these ‘hot spots’ are linked to pathogens other than *Plasmodium* spp. [9]. At least 15 zoonotic pathogens capable of causing febrile illness have been historically reported in Namibia’s 13 administrative regions [11]. Pathogenic bacteria, notably *C. burnetii*, *Bartonella henselae*, as well as, spotted fever and typhus group rickettsiae were highlighted as potentially posing the greatest risk to animal and human health [11]. These zoonotic bacteria have been increasingly noted throughout sub-Saharan Africa, and there is a need to specifically focus on their epidemiology [12]–[14].

*Coxiella burnetii*, the causative agent of Q fever, is potentially transmissible via blood transfusion [15], [16] or even by ticks [11]. Reported to affect pregnant woman, *Coxiella burnetii* can cause foetal loss and affect intrauterine growth of the foetus [13]. Spotted fever and typhus group rickettsiae are also common causes of febrile illness in sub-Saharan Africa [17]–[22]. Transmitted mainly by ticks and fleas [23], the pathogenesis of rickettsial infections can involve a spectrum of conditions, from asymptomatic infections to death [18]. *Bartonella henselae* (cat scratch disease) is usually associated with the bite of an infected flea [14], [24] and has been reported to affect immune-compromised persons such as HIV-infected persons or those with heart valve issues [25]–[27]. Because of the undifferentiated symptoms often associated with Q fever or rickettsial infections, a lack of specific diagnostic tests frequently has led to misidentification with each other [28] or arboviruses [29]–[31].

Rickettsial diseases associated with spotted fever and typhus group rickettsiae as well as Q fever, and cat scratch disease have been recorded in neighbouring countries (South Africa [17]; Botswana [32]; Zambia [33]; Angola [34] and Zimbabwe [35]. However, virtually nothing is known about their presence in Namibia [11]. A single small serosurvey in northern Namibia in the mid-1980s reported a 2% seroprevelence for *C. burnetii* and evaluated the presence of rickettsiae, but the diagnostics used were not conclusive [36]. No studies have evaluated the presence of *B. henselae* in Namibia.

To evaluate possible zoonotic bacteria involved with non-malaria febrile illness in Namibia, this study assessed the current exposure of a healthy volunteer cohort to these four zoonotic bacteria and attempted to determine possible risk factors through a questionnaire. Volunteer blood donors are commonly used to evaluate exposure to bacterial pathogens [34], [37]–[40], however, prevalence estimates from these studies are considered conservative because blood donors are normally younger, healthier and screened for other significant pathogens [41], [42]. Volunteer blood donor samples are often used as controls in studies evaluating occupational risk of exposure to zoonotic pathogens [43]–[45].

## Methods

### Ethical statement

The study was approved by the Permanent Secretary and the Research Committee of the Ministry of Health and Social Services (MoHSS) of Namibia, the Institutional Research and Publications Committee of the Polytechnic of Namibia as well as the NAMBTS leadership. All blood donors who agreed to participate signed their consent form and their personal information was held by NAMBTS. The research team (including the NAMBTS personnel involved) only received the completed (and de-identified) surveys and two vials of donor serum, both of which were linked to a unique donor identification number.

### Study population and study design

A purposive cross-sectional sample of volunteer blood donors was collected between September 2011 and February 2012. Donor specimens were collected by the Blood Transfusion Service of Namibia (NAMBTS) from fixed and mobile donation clinics. The samples collected were as part of a broad sero-survey for viral, bacterial and protozoan zoonotic pathogens in Namibian volunteer blood donors. The number of volunteers enrolled (n = 319) was established with EpiInfo 6.0 (CDC, Atlanta, GA, USA), using an estimated prevalence of 30% (based on *C. burnetii* exposure in Zimbabwe [35], an absolute error of 0.5 and a 95% confidence interval (95%CI).

### Sample selection

Inclusion criteria included healthy individuals (first time or repeat donors) who passed the donor selection criteria set by NAMBTS [46]. Before donating blood, each volunteer was provided a study consent form in English or Afrikaans. Due to the focus of the study, we attempted to over-sample volunteer blood donors with rural or farm experience. After reading and discussing the form with NAMBTS staff, each volunteer was asked whether they currently lived in a farm or rural area, or had considerable exposure to farm or rural areas in Namibia during the past 10 years. If affirmative, donors were asked if they wanted to participate in this study. If they agreed, they signed the consent form, completed a short questionnaire (in English or Afrikaans) and an additional 4 ml of blood was drawn during their donation session.

### Serum samples

After donation, blood samples were transported to the NAMBTS headquarters in Windhoek where they were processed and serum components were divided into two 2 ml vials, each labelled with a unique patient identification number, and stored at −20°C until picked up for testing at the Polytechnic of Namibia.

### Questionnaire

The questionnaire included questions on demographics such as gender, age, region, and the general area where they lived (city, peri-urban or rural). Questions focused on donors’ interactions with animals (types, frequency, occupational), experience living on farms or in rural areas, memory of tick bites that took more than a week to heal, unexplained fevers following periods of exposure to the “veldt” (farm or rural area), and histories of ‘tick-bite fever’ either personally or in a household pet over the past 5–10 years. These variables were selected based on similar studies in the literature.

### Serological testing

Serological testing on the serum samples was performed in the Department of Biomedical Science at the Polytechnic of Namibia using the following IgG (Immunoglobulin G) antibody kits from Fuller Laboratories (Fullerton, California, USA): 1) MIF IFA (micro-immunofluorescence assay) IgG antibody (gamma chain specific) kits for *C. burnetii* (Phase 1 and 2 in the same IFA spot); 2) ELISA (enzyme-linked immunosorbent assay) IgG antibody (gamma chain specific) kits for rickettsiae groups (spotted fever and typhus group rickettsiae); 3) MIF IFA IgG antibody (gamma chain specific) kits for *Rickettsia africae*/*R. typhi* (in same IFA spot); and 4) IFA (immunofluorescence assay) IgG antibody (gamma chain specific) kits for *B. henselae*. Testing protocols followed manufacturer’s instructions, including the cutoff calibrator instructions for the rickettsiae ELISA. All IFA slides were screened by two trained persons using a Primo Star iLED fluorescence microscope (Zeiss), recommended for developing countries by the WHO [47]. Only samples that had a questionnaire were included in the study. Methods for each test kit include the following:

#### *Coxiella burnetii*

Initial screening (1∶16 dilution) was followed by confirmation screening (1∶256 dilution). Each commercially-prepared slide contained 12 spots containing acetone-fixed cells containing phase I and II *C. burnetii*. Briefly, ten microliters of diluted patient serum was added to each spot. Manufacturer-prepared positive and negative controls were run with each cohort of serum tested. After incubation at 37°C for 30 minutes and subsequent PBS washing, ten microliters of conjugate (affinity-purified DyLight 488-labeled goat anti-human IgG (heavy chain) with bovine serum albumin) and Evans’ blue counterstain was added. After incubation and washing, mounting medium was added prior to reading under fluorescence microscope. Only IFA spots which rated 2+ or higher in fluorescence intensity were considered positive (test kit instructions). Each positive or equivocal *C. burnetii* result was re-confirmed.

#### Spotted Fever and typhus group rickettsiae

All samples were screened at a 1∶100 dilution using an antigen-specific ELISA. The Spotted Fever Rickettsia IgG EIA Antibody kit used a group-specific lipopolysaccharide antigen that cross-reacts among spotted fever group rickettsiae species and the *Rickettsia typhi* EIA IgG Antibody kit used a species-specific Outer Membrane Protein B antigen which also cross-reacts among other typhus group rickettsiae. Briefly, 100 µl of diluted serum, negative control and dilutions of positive control serum were pipetted into antigen-coated 96 well plates. After incubation and washing with a PBS/Tween buffer, 100 µl of affinity-purified HRP-labeled goat anti-human IgG (heavy chain) was added to each well. After incubation and washing, 100 µl of a tetramethylbenzidine (TMB) solution was added to each well for 10 minutes prior to adding a stop solution. Absorbance was read using a microplate reader with a 450 nm filter. For quality control, the kit contained a cutoff calibrator to discriminate between reactive and non-reactive samples. All ELISA positive and equivocal samples were re-confirmed using MIF IFA [48] containing *R. africae* and *R. typhi*-infected Vero cells which utilized a similar protocol described above for *C. burnetii* with appropriate positive and negative controls. While the positive controls worked for the *R. africae* (spotted fever group rickettsiae), the *R. typhi* controls did not work even after replacement of the kits. As such, only the IFA results for spotted fever group rickettsiae confirmations are presented. The data used for risk factor analysis was based on the ELISA results for both groups.

#### *Bartonella henselae*

Screening and rescreening of exposure was carried out at a 1∶64 dilution using a *Bartonella henselae* IFA human IgG Antibody kit. Each commercially-prepared slide contained 12 spots of *B. henselae*-infected Vero cells. Similar to the *C. burnetii* protocol, ten microliters of diluted patient serum was added to each spot and manufacturer-prepared positive and negative controls were run with each cohort of serum. After incubation and washing, ten microliters of conjugate and mounting medium was added after incubation/washing and mounting medium was added prior to reading under fluorescence microscope. Only IFA spots which rated 2+ or higher in fluorescence intensity were considered positive (as per test kit instructions). Due to funding constraints, only 105 samples were tested, specifically targeting those donors who had indicated a frequent interaction with dogs or cats, a common risk factor [14].

### Statistical analysis

All data were entered into Microsoft Excel spreadsheets and analyzed using SPSS (version 21, IBM Corp., Armonk, NY, USA). Pearson’s χ^2^ tests or Fisher’s exact tests (when values were lower than 5) were used for categorical data. Bivariate analyses assessed associations between population characteristics provided in the surveys and seropositivity results. Odds ratios (OR) and 95% confidence intervals (CI) were calculated for all associations. P values less than 0.05 were considered statistically significant.

## Results

### Study population

Serum samples (n = 319) were collected by NAMBTS, but 14 did not have accompanying surveys and were excluded. While not all 305 samples were tested for all four pathogen groups, each sample was tested for exposure to at least one pathogen in the study. The mean age of donors was 32.2 years±11.3 years (range 18–64). Almost half of the donors were under the age of 30 (46.1%) and 86.8% of the donors were from the central regions of Namibia (Otjozondjupa, Erongo, Khomas, Omaheke) (Table 1). Eleven of Namibia’s 13 regions were represented in the study sample, even if by only one specimen (Caprivi, Ohangwena, Omusati). No specimens were available for Kavango and Karas regions. Most of blood donors (70.1%) currently lived, or had lived, in rural area or on a farm with exposure to animals in the past 10 years. The majority (81.4%) of those tested lived in urban settings and interacted regularly with animals (97.3%) (domestic and companion animals).

**Table 1:** Characteristics of the Namibian study population and risk factors associated with exposure to *C. burnetii*, spotted fever and typhus group rickettsiae.

	Study population		*Coxiella burnetii* (1∶16 dilution)		spotted fever group (1∶100 dilution)		typhus group (1∶100 dilution)	
		N (%)	N (%)	OR (95% CI)	N (%)	OR (95% CI)	N (%)	OR (95% CI)
Gender	Females	106 (35.9%)	19/98 (19.4%)	0.57 (0.31–1.03)	5/95 (5.3%)	0.3 (0.11–0.81)*	7/95 (7.4%)	0.34 (0.14–0.80)*
	Males	189 (64.1%)	53/178 (29.8%)	1	27/174 (15.5%)	1	33/174 (19.0%)	1
Age groups	Under 20	34 (11.5%)	3/33 (9.1%)	0.35 (0.07–1.78)	1/31 (3.2%)	0.17 (0.02–1.74)	3/31 (9.7%)	0.21 (0.05–0.99)*
	20–29	102 (34.6%)	27/95 (28.4%)	1.39 (0.42–4.60)	12/93 (12.9%)	0.74 (0.19–2.94)	11/93 (11.8%)	0.27 (0.84–0.86)*
	30–39	88 (29.8%)	24/81 (29.6%)	1.47 (0.44–4.94)	8/79 (10.1%)	0.56 (0.13–2.38)	13/79 (16.5%)	0.39 (0.12–1.24)
	40–49	50 (16.8%)	14/49 (28.6%)	1.40 (0.39–4.99)	8/48 (16.7%)	1.0 (0.23–4.28)	7/48 (14.6%)	0.34 (0.09–1.21)
	50 and above	21 (7.1%)	4/18 (22.2%)	1	3/18 (16.7%)	1	6/18 (33.3%)	1
Areas∧	North	22 (7.5%)	3/20 (15.0%)	.016 (0.03–0.74)*	5/20 (25.0%)	5.0 (0.52–48.07)	2/20 (10.0%)	0.33 (0.05–2.11)
	Central	256 (86.8%)	60/239 (25.1%)	0.30 (0.11–0.81)*	26/233 (11.2%)	1.88 (0.24–14.86)	34/233 (14.6%)	0.51 (0.16–1.68)
	South	17 (5.8%)	9/17 (52.9%)	1	1/16 (6.3%)	1	4/16 (25%)	1
Residence	Rural	55 (18.6%)	17/53 (32.1%)	1.5 (0.79–2.86)	9/53 (17.0%)	1.62 (0.71–3.75)	7/53 (13.2%)	0.8 (0.33–1.9)
	Urban	240 (81.4%)	53/217 (24.4%)	1	23/210 (11.0%)	1	32/210 (15.2%)	1
Worked with animals on farm?	No	179 (60.7%)	33/161 (20.5%)	.05 (0.29–0.86)*	17/160 (10.6%)	0.74 (0.36–1.56)	21/160 (13.1%)	0.72 (0.36–1.4)
	Yes	116 (39.3%)	39/115 (33.9%)	1	15/109 (13.8%)	1	19/109 (17.4%)	1

*Statistically significant at P<0.05.

∧ Regions: North (Caprivi, Ohangwena, Oshikoto, Oshana, Omusati, Kunene); Central (Otjozondjupa, Erongo, Khomas, Omaheke); South (Hardap).

### *Coxiella burnetii*

Of the 276 samples tested by IFA for exposure to C. burnetii (1∶16 dilution), 26.1% (n = 72) were positive for antibodies to either Phase I or Phase II antibodies. Thirty-four (12.3%) and 66 (23.9%) of samples tested were positive for Phase 1 or Phase II antigens, respectively. Twenty-seven (37.5%) samples were positive for both phases. Of the samples with higher titers to Phase I antigens (≥1∶256), seven (20.6%) were positive compared with 15 (22.7%) of the samples with higher titers to Phase II antigens (≥1∶256).

For the 276 samples tested for exposure to *C. burnetii*, being male, living in Hardap region (southern Namibia), and regularly working with animals (hunters or farmers) were significant risk factors (Table 1), while age, urban/rural residence, and occasional interaction with animals were not significant. When evaluating specific animal groups, those interacting with cattle, donkeys and/or horses were significantly at risk for exposure (OR = 2.2 (95%CI 1.2–3.9; P<0.007). Interactions with other animals (dogs, cats, sheep, goats, and wild animals) were not significant.

### Spotted fever group rickettsiae

Of 269 samples tested, thirty-two (11.9%) samples were ELISA positive for spotted fever group rickettsiae. Twenty-three (71.9%) of the 32 ELISA positive samples were confirmed by IFA using *R. africae*-infected cells. Being male was the only significant risk factor, while urban/rural residence, age, region, and interactions with animals were not risk factors (Table 1). While not significant, there may be a regional trend in exposure, with higher rates in northern regions and the lowest prevalence in the southern region of Hardap (Table 1).

### Typhus group rickettsiae

Of 269 samples tested, 40 (14.9%) samples were ELISA positive for typhus group rickettsiae. Being male and in the 20–29 year old age group were the only significant risk factors, while urban/rural residence, region, and interactions with animals were not significant (Table 1). Similar to *C. burnetii*, while not significant, there may be a regional trend in exposure with lower rates in the northern regions and higher rates in the south (Table 1).

### Multiple pathogen exposure

Of the 268 patients tested for exposure to all three pathogens (*C. burnetii*, spotted fever and typhus group rickettsiae), 117 (43.7%) had antibodies indicative of exposure to at least one of the pathogens. The majority (78.6%) were positive for only one pathogen with 55 (47%) reacting with only *C. burnetii* while 23 (19.7%) and 14 (12%) reacting only with spotted fever group and typhus group rickettsiae, respectively. One in five blood donors (20.4%) had been exposed to two or more pathogens with 8 (6.8%) samples recognizing each combination (typhus and spotted fever group rickettsiae, *C. burnetii* and typhus group rickettsiae, *C. burnetii* and spotted fever group rickettsiae). Only one sample reacted with all three pathogen/groups.

### *Bartonella henselae*

Three (2.9%) of 105 samples tested for exposure to *B. henselae* were positive. These three consisted of two males and one female from Central regions in Namibia. Two lived in rural areas. Only one had a dog or cat. One was also positive for *C. burnetii* Phase II antibodies.

### Questionnaire

Of the 305 questionnaires reviewed, 39.7% of the respondents had an occupation in which they interacted regularly with animals (mainly farming or hunting) and two were veterinarians. Seventeen (5.8%) recalled a tick-bite that took longer than a week to heal and ten (3.4%) recalled an unexplained fever a few days after being in the veldt. Forty-two (14.2%) owned an animal recently diagnosed with ‘tick-bite fever’. However, when analysed in conjunction with exposure to *C. burnetii* and spotted fever and typhus group rickettsiae, only one response variable was significant. Among those exposed to *C. burnetii*, 40% (16/40) had a pet recently diagnosed with ‘tick-bite fever’ compared with 22.9% (52/227) who did not have a pet recently diagnosed with ‘tick-bite fever’ (OR = 2.2 (95%CL 1.1–4.5) (P≥0.03).

Four (1.4%) donors surveyed had been diagnosed with ‘tick-bite fever’ by a medical professional. Each donor was positive for exposure to a different pathogen or combination of pathogens: 1) typhus group rickettsiae; 2) spotted fever and typhus group rickettsiae; 3) *C. burnetii*; and 4) typhus group rickettsiae and low levels of *C. burnetii* antibodies.

## Discussion

This was first study since Namibian Independence (1990) to describe exposure to *C. burnetii*, spotted fever and typhus group rickettsiae and *B. henselae* in a cohort of healthy Namibians. The results are comparable with past studies in the Southern African region. The observed 26% prevalence for *C. burnetii* is comparable with the 37% (n = 494) reported in Zimbabwe [35] but quite different that the 2% (n = 113) from Angola [34]. The spotted fever group rickettsiae prevalence of 12% among the donor samples was considerably lower than the 55% (n = 276) reported in Zimbabwe [36], 48% in Angola [34], but similar to the 17% (n = 377) reported in Zambia [33]. The 15% prevalence to *R. typhi* can only be compared with 5% reported in Zambia [33] and 0% in Angola [34]. Recent *B. henselae* prevalence rates in South Africa were recently reported at 23% (n = 382) in HIV-infected persons and 10% (n = 42) in clinically healthy volunteers [49] which is considerably higher than the 3% found in the current study.

The global distribution of *C. burnetii* and its effect on human and animal health is well reviewed [13], [50], [51]. Because zoonotic disease is usually spread to humans via infected livestock [13], the significant regional trend observed between northern (15%) to southern (52%) areas in Namibia could be important as a guide to future public health or disease prevention and control efforts. While the majority of samples were from central regions, the high seroprevelence among apparently healthy blood donors from the southern Hardap region (9 of 15– most of them youth) could indicate a significant transmission zone in Hardap. This finding could potentially impact human and animal health and guide future public health interventions.

Like numerous studies worldwide [40], [43], [52], persons who worked regularly with animals (OR = 2.0), particularly cattle, donkeys and horses (OR = 2.2), may be more at risk in Namibia. *Coxiella burnetii* has a broad transmission potential especially among occupations that involve inhalation of small cell variants and aerosols or ingestion of unpasteurized milk [13]. While generally noted that chronic *C. burnetii* infections in cattle, sheep and goats may cause reproductive issues such as abortion, premature, dead or weak offspring, cattle are more often affected than other livestock species [51]. Because most recorded livestock cases in Namibia have occurred in goats [53], the findings of the present study could further focus public health interventions to prevent animal and human infections.

It was surprising to find a significant association between exposure to *C. burnetii* and a pet recently diagnosed with ‘tick-bite fever’, most likely due to *Ehrlichia canis*, *Hepatozoon canis*, or *Babesia canis vogeli* [54]. While the effect of *C. burnetii* on the reproductive health of companion animals is not well defined [51], dogs and cats are known reservoirs for *C. burnetii* in Zimbabwe [35], [55]. Future studies should follow up this possible link between companion animal health and *C. burnetii* exposure as well the possibility of cross-reactivity to *Bartonella* species [56].

*Rickettsia africae* has only been reported in Namibia from unspecified tourist travel studies [18]. While its presence in neighbouring countries indicates a probable presence in Namibia [17], [19], [33], no study has yet addressed the likelihood of spotted fever group rickettsiae among Namibians. Gender was the only significant risk factor involved with males (OR = 3.3) more likely to be exposed as they are possibly more active outdoors on farms or rural areas.

While the study did not differentiate between rickettsial species, spotted fever rickettsiae are normally transmitted by ticks or fleas, of which eight potential vector species have been recorded in Namibia [11]. *Rickettsia felis*, a spotted fever group rickettsiae transmitted by cat or rat fleas [57], remains to be identified south of Angola, Zambia and Mozambique. To date, the most southerly report in sub-Saharan Africa is from the Democratic Republic of Congo [58]. Given the long history of ports in Southern Africa and the association of *R. felis* with rat and cat fleas, it is likely a matter of being underreported. The results of this study demonstrate the presence of spotted fever group rickettsiae in Namibia but indicate the need to follow up with molecular identification in order to establish the current epidemiology and confirm the southern to northern trend in exposure which could involve the species composition of vector ticks.

Like spotted fever group rickettsiae, 15% of samples were positive for typhus group rickettsiae, with a significant association with males under 30 years of age, the majority of whom did not regularly work on farms or hunt. The two principal *Rickettsia* spp. possibly involved are *R. prowazekii* (louse-borne) or *R. typhi* (flea-borne) [57]. While *R. prowazekii* (endemic typhus) is probably not present due to microhabitat conditions and lack of non-migrating populations, *R. typhi* is most probable. The question, however, of how a pathogen normally found in urban or port cities [57] could be prevalent in a dry, arid climate in Central Namibia needs follow up. The 7–10% seroprevalence of *R. typhi* among cats in similar climates of Zimbabwe and South Africa indicates that climate may not significantly limit the epidemiology [59]. The observed north-south regional trend observed may not be important, either, as Wessels et al. [36] observed a low agglutination response in 59% of patients in the northern Kavango region, indicating possible exposure to typhus group rickettsiae [11]. Compared with other countries in Southern Africa, the relatively high prevalence among Namibian blood donors demonstrates a need for further follow up on this important pathogenic group.

This is the first report of possible exposure to *B. henselae* in Namibia. The low seroprevalence (3%) among blood donors is lower than other studies among healthy volunteers in South Africa (10%) [49]. Also known as ‘cat scratch disease’, *Bartonella henselae* appears to be common in Southern Africa with prevalence rates of 21–23% reported among domestic and wild felines [60] and 14% in dogs [61] in Zimbabwe and South Africa. While the low prevalence in the study population indicates the presence of *Bartonella* spp. in Namibia, lack of exposure to infected fleas or the dry, arid climate may reduce actual risk among residents in the central regions. Future studies should evaluate the presence of *B. henselae* in fleas, companion animals, and potentially patients with undifferentiated febrile illness in the coastal areas around the cities of Luderitz, Walvis Bay and Swakopmund. As in other countries, cross-reactivity could have occurred with other *Bartonella* spp. that may be present in the region [17] as well as *C. burnetii* [48].

One of five samples was positive for exposure to two or more pathogens. These results probably indicate that persons tested had either experienced an active infection or had an exposure to the pathogen which was significant enough to stimulate antibodies. While the ELISA kits used were based on antigens which differentiated between spotted fever and typhus group rickettsiae [62], [63], cross-reactivity has been recorded among spotted fever rickettsiae species [64] and between *Bartonella* and *C. burnetii* [56].

The exposure to bacterial pathogens among volunteer blood donors was a significant component in this study as it may be important for the safety of the national blood supply. While it is unlikely that spotted fever and typhus group rickettsiae would survive the blood storage process [65], *B. henselae* [15] and *C. burnetii* [16] are known to survive in donated blood units and can be transmitted via transfusion. A transfusion of *C. burnetii* into an immunocompromised person, either pregnant or HIV-infected (19% HIV-infection rate in Namibia [66]), could result in an abortion in the pregnant woman or death of the HIV-patient.

One exciting aspect of this study was the use of a Primo-star iLED scope for determination of IFA results. The Primo-star iLED microscope was designed for research laboratories in resource-limited settings, where cost, efficiency, and ease of use are important [67]. This microscope has been shown to have equivalent accuracy to that of international standards and is now recommended by the World Health Organization as a standalone option for field diagnosis of tuberculosis [47].

In regards to limitations, it is difficult to identify true regional trends and establish risk factors from a small, non-random sample. Also, because most Namibian blood donors are males with farm/rural experience from the central regions, it was not possible to gather a more heterologous population for a true population-based epidemiological assessment. The cross-sectional approach to use only one sample from each donor meant that the benefits of comparing acute and convalescent samples from febrile inpatients were not present [3]. While these limitations require a broader, population-based investigation in order to potentially impact public health policy, the study demonstrated that these pathogens are present among healthy persons and raises the profile of the issue of vector-borne bacterial pathogens as a source of non-malaria fevers. Without Western Blot, molecular confirmation of DNA present in the blood, or cultivation of viable pathogen from specimens, this study can only state broadly that Namibians are being exposed to these groups of pathogenic bacteria. Additionally, samples were only screened at 1∶16 and 1∶256 titers for antibodies to *C. burnetii*, and 1∶64 for *B. henselae* but end point titers were not determined. While indicating a rather high seroprevalence of exposure to *C. burnetii*, future work is needed to fully characterize the serologic status of the population in regards to possible acute and chronic infections.

## Conclusions

This study establishes that Namibians, particularly those involved in farms or rural life, are being exposed to pathogenic, vector-borne bacteria. Given Namibia’s priority to eliminate malaria [9], it will not be possible to upgrade national diagnostic algorithms to account for other causes of febrile illness without active clinical surveillance. While most of these bacteria can be identified via symptoms and simply treated using tetracycline-class antibiotics, it is critical for health professionals to begin differentiating between these four types of pathogens. The possibility of missing an acute infection that could become chronic could cause serious complications for either the patient (*R. conorii*) [29], the foetus of an infected pregnant woman [13] or a person receiving a blood transfusion (*C. burnetii*) [16]. The low prevalence of *B. henselae* among blood donors may actually be a positive finding, indicating that this pathogen may not be as important as Q fever and rickettsiae in the health of Namibians.

Knowing that healthy Namibians are encountering these pathogens, future studies should concentrate on three main areas: 1) Determine the frequency of febrile illnesses caused by vector-borne bacterial pathogens; 2) Further describe the occupational risks factors for different professions, such as farmers, abattoir workers, and veterinarians, and; 3) Systematically map the occurrence of each pathogen in humans and animals, determine the risk of transmission by different vector species, and develop public health interventions to reduce vector populations or transmission. Without vectors, most of these bacterial pathogens (except *C. burnetii*) will not spread [14].
